# Mutation SVCT2 promotes cell proliferation, invasion and migration in colorectal cancer

**DOI:** 10.7150/jca.57463

**Published:** 2021-07-06

**Authors:** Sang-Soo Park, Yea Seong Ryu, Dong-In Koh, Seung-Woo Hong, Jai-Hee Moon, Jae-Sik Shin, Mi Jin Kim, Do Yeon Kim, Jun Ki Hong, Eun Ho Kim, Hong-Rae Jeong, Yoon Sun Park, Joseph Kim, Dong Min Kim, Hyeseon Yun, Joo-Yeon Shin, Dong-Hoon Jin

**Affiliations:** 1 Asan Institute for Life Science, Asan Medical Center, Seoul, Republic of Korea; 2 Department of Medical Science, Asan Medical Institute of Convergence Science and Technology, Asan Medical Center, University of Ulsan College of Medicine, Seoul, Republic of Korea; 3 Department of Convergence Medicine, Asan Medical Center, University of Ulsan College of Medicine, Seoul, Republic of Korea

**Keywords:** SVCT2, colorectal cancer, cell proliferation, invasion, migration

## Abstract

The sodium-dependent vitamin C transporter 2 (SVCT2) surface glycoprotein regulates ascorbate accumulation in the plasma, often resulting in the induction of cancer cell death. Therefore, high expression of this gene associates with increased overall survival in several cancers. However, in colorectal cancer (CRC), high (likely mutated) *SVCT2* expression relates to poor overall survival, and its functional significance has not been studied. Thus, we hypothesize that mutant *SVCT2* expression could affect CRC patient survival. According to biological databases, *SVCT2* has been found to be mutated frequently, and *SVCT2* E264K has a particularly high pathogenic score (0.98), compared to other *SVCT2* mutant sites, in CRC patients. Interestingly, our results reveal expression of *SVCT2* E264K in many CRC tissues and cells. Also, we found wild-type SVCT2 expression to be largely localized to the cytoplasm and membrane, while SVCT2 E264K was restricted to the cytoplasm. We further found that *SVCT2* E264K overexpression increases cell growth. By contrast, *SVCT2* E264K knockdown significantly reduced cell proliferation and promoted cell apoptosis, resulting in inhibition of cell invasion and migration. Taken together, SVCT2 E264K plays a critical role in proliferation in CRC. Our results suggest that SVCT2 E264K could be a promising novel therapeutic target in CRC.

## Introduction

Colorectal cancer (CRC) is the third-most common cancer worldwide, and its incidence and mortality are steadily rising in Asia, Europe, and North and South America 1, 2. According to GLOBOCAN, CRC is the second-leading cause of global mortality, accounting for an estimated 9.6 million annual deaths 2. Accumulating evidence has shown that early cancer stage, i.e., stage 1 CRC has a 5-year survival rate of over 90%; in stage 4, however, such survival drops to ~10% 3. Furthermore, about 25% of total CRC patients still develop symptoms from the disease or its metastasis 4-6.

Genetic alterations in the colonic epithelium drive from transformation of normal epithelium to an adenoma, progressing to in situ carcinoma, and eventually, to an invasive and metastatic tumor 7. CRC is a genetically heterogeneous disease involving a vast array of mutations, suggesting that targeted therapy, for specific molecular aberrations, is likely to be effective for patients 8. Importantly, it has become obvious that developments in molecular staging add a source of prognostic and predictive information, to enhance the classic staging system, in which CRC patients are classified into four different prognostic groups, based on the extent of the primary tumor, the involvement of regional lymph nodes, and the presence/absence of metastasis 8. Therefore, it is of significant importance to identify clinical biomarkers responsible for monitoring CRC treatment, which may further lead to the development of novel therapeutic targets, ultimately decreasing the risk of death in CRC patients.

Sodium-dependent vitamin C transporters (SVCTs) are surface glycoproteins, encoded by two different genes, having very similar structures, while also showing distinct functional properties, depending on their cellular distribution 9-11. *SVCT1* is primarily expressed in epithelial tissues such as the intestine, liver, and kidney, while *SVCT2* is ubiquitously expressed in a wide variety of tissues, including the placenta, liver, brain, heart, lung, intestine, and eye 12-18. With regard to function, SVCT1 is a low affinity and high capacity vitamin C transporter that responds to most ascorbate transporters, whereas SVCT2 is a high affinity and low capacity vitamin C transporter that regulates ascorbate accumulation in the plasma 19-21. Although most animals can synthesize their own vitamin C in the liver, homozygous *SVCT2* knockout mice die within a few minutes after birth, due to brain hemorrhage and respiratory failure 22 indicating that this transporter is required for maintaining vitamin C homeostasis.

According to Kaplan-Meier survival analysis, high *SVCT2* expression associates with good prognosis in several cancers, including liver, pancreatic, and urothelial cancer, but not in CRC. Consequently, we hypothesize that mutant SVCT2 expression could be overexpressed and even affect survival in CRC. Mutations or DNA methylation in a tumor suppressor gene results in a loss or reduction in its function and often upregulation and activation of oncoproteins 23. According to the Catalogue of Somatic Mutations in Cancer (COSMIC) database, *SVCT2* has been found to be mutated frequently and *SVCT2* E264K, specifically, has a pathogenic score of 0.98, distinctly higher than other *SVCT2* mutant sites, in CRC patients. However, the functional significance of *SVCT2* E264K has not been studied. This finding suggests that previous studies showing *SVCT2* overexpression, in tumors, could have actually been high expression of a mutant *SVCT2* gene (similar to the case of the tumor suppressor p53) (PMID: 2137806). It was also reported that the missense mutant *SVCT2* results in decreased amino acid uptake, and consequent inhibition of osteoblast-like differentiation in murine calvarial MC3T3-E1 cells 24. Furthermore, genetic variations in *SVCT2* associate with increased risk of numerous malignancies, including gastric cancer, lymphoma, and head and neck squamous cell carcinomas 25-27. Therefore, it is important to consider the molecular characterization of mutant *SVCT2* E264K for CRC patient prognostic and therapeutic assessment.

In this study, we examined functional differences between wild-type *SVCT2* and *SVCT2* E264K in CRC. Moreover, we investigated the mechanisms underlying mutant form *SVCT2*-induced cell proliferation, thus assessing its importance as a novel therapeutic target for CRC treatment.

## Materials and methods

### Cell culture

Human CRC HCT 116, HCT-15, COLO 320DM, HT-29, DLD-1, Caco2, SW480, SW480E, SW620, and RKO cell lines were purchased from the Korean Cell Line Bank (KCLB, Seoul, Korea). Human embryonic kidney 293T cells were obtained from American Type Culture Collection (ATCC, Manassas, VA, USA). Cell lines were cultured in RPMI 1640 (Welgene, Daegu, Korea) or DMEM (Welgene) medium containing 10% heat-inactivated fetal bovine serum (Gibco, Thermo Fisher Scientific, Waltham, MA, USA) at 37°C in an incubator with a humidified atmosphere of 5% CO_2_.

### Patients and specimens

Tumor samples from a total of 64 patients were used in this study. Clinical samples were provided by ProteoGenex, Inc. (Inglewood, CA, USA).

### Kaplan-Meier Plotter tool analysis

The Kaplan-Meier Plotter database (GEPIA) was used to determine the association between *SVCT2* and overall survival in CRC patients. *SVCT2* mRNA expression was classified as 'high group' and 'low group' according to the gene expression values with preestablished cutoffs. The Kaplan-Meier survival plot, hazards ratio (HR), 95% confidence interval (CI), and log rank p were directly determined and exhibited on the web page. *p* value less than 0.05 was considered statistically significant.

### Transfection of plasmids and short hairpin RNAs (shRNAs)

Cells were transiently transfected with wild-type *SVCT2*-, and *SVCT2* E264K-expressing plasmids, and shRNA against both mRNAs using jetPRIME reagent (Polyplus-transfection SA, Illkirch-Graffenstaden, France), according to the manufacturer's instructions. The integrity of the constructs was confirmed by sequencing. Targeting the *SVCT2*-coding region with shRNA was used to silence gene expression. Negative control scrambled shRNA, with no significant homology to human gene sequences, was used to control for nonspecific effects.

### Quantitative reverse transcription polymerase chain reaction (qRT-PCR)

Total RNA was extracted using TRIzol reagent (Invitrogen, Carlsbad, CA, USA), and 1 μg RNA was reversed transcribed using the AccuPower^®^ RT PreMix (Bioneer, Daejeon, Korea). PCR was then performed using AccuPower^®^ Gold Multiplex PCR PreMix (Bioneer) in a ABI 9902 thermal cycler (Applied Biosystems, Foster City, CA, USA). The primers for PCR products were as follows: using the forward primer 5'-CATCGGTCCCTTGACCATTAC-3', and the reverse primer 5'-GTTGGGCTGATGGGTAAGTAG-3', for amplification of fragment #1; the forward primer 5'-CCAAGTTACCTCAGACAGAACC-3', and the reverse primer 5'-CCAGCAATGGACACTCTCAA-3', for the amplification of fragment #2; the forward primer 5'-CATCGGTCCCTTGACCATTAC-3', and the reverse primer 5'-ACCGTGAAGATGAAGCAGAG-3', for the amplification of fragment #3. PCR for all four amplicons were as follows: 5 min at 95°C, for initial denaturation; 35 cycles of 30 sec at 95°C, 30 sec at 58°C, and 45 sec at 72°C, and final elongation for 5 min, at 72°C. The PCR products were then resolved by 2% agarose gel electrophoresis.

### Subcellular fractionation

Cytoplasmic and membrane fractions were isolated using Subcellular Protein Fractionation Kits for Cultured Cells (Thermo Fisher Scientific), following the manufacturer's instructions. In brief, cell pellet was mixed with ice-cold CEB buffer including protease inhibitors and incubated at 4°C for 10 min. Insoluble material was sedimented at 500 × *g* for 5 min and the resulting supernatant, the cytoplasmic extract, was transferred to a clean pre-chilled tube on ice. The pellet was mixed with ice-cold MEB buffer containing protease inhibitors and incubated at 4°C for 10 min. The insoluble component was sedimented at 3,000 × *g* for 5 min. The supernatant (membrane extract) was transferred into a clean pre-chilled tube on ice. Fractions were stored at -80°C until further use.

### Western blotting

Total proteins were extracted, separated by 10-15% sodium dodecyl sulfate-polyacrylamide gel electrophoresis (SDS-PAGE), and transferred to 0.45 μm polyvinylidine difluoride (PVDF) membranes (Millipore, Billerica, MA, USA). The membranes were incubated with 5% skim milk to block nonspecific binding at room temperature for 1 h, and then incubated with primary antibodies overnight at 4°C, including anti-SVCT2 from Abcam (Cambridge, MA, USA). Antibodies against Myc-tag, phospho-Akt, Ak, Survivin, phospho-Erk, Erk, and E-cadherin were from Cell Signaling Technology (Danvers, MA, USA), while antibodies against α-Tubulin, β-Actin and Gapdh were from Santa Cruz Biotechnology (Santa Cruz, CA, USA). The membranes with bound primary antibodies were then reacted with horseradish peroxidase (HRP)-conjugated secondary antibodies (Santa Cruz) for 2 h at room temperature, and the protein bands then detected by chemiluminescence (Amersham, Little Chalfont, Buckinghamshire, UK). Antibodies against α-Tubulin, β-Actin and Gapdh were used as loading controls. The intensity of the bands was quantified by Image J software.

### Proliferation assay

Following cell transfection, 1 × 10^5^ cells were seeded in 6-well plates, for the indicated times. First, the cultured cells were prepared into single cell suspensions by trypsin solution. The cells were then pipetted up and down to obtain single cell suspensions and mixed with an equal amount of 0.4% trypan blue stain (Gibco-BRL, Thermo Fisher Scientific). Since viable cells maintain membrane integrity and do not take up trypan blue, stained (dead) cells were counted under a light microscope 28. These assays were repeated at least three times.

### Colony formation assay

Following cell transfection, cells were seeded in 6-well plates (500 cells/well) for a ≤ 2-week incubation, which was terminated when white clone spots were observed by the naked eye. The colonies were then fixed with 100% methyl alcohol, and stained with 0.05% crystal violet for 20 minutes at room temperature. Colonies containing 50 or more cells were considered viable.

### Microscopic examination

To examine morphological changes, 1 × 10^5^ cells were seeded in 6-well plates and transfected with *SVCT2* E264K. The cells were rinsed twice with PBS and an inverted phase-contrast microscope (EVOS® FL Cell Imaging System, Thermo Fisher Scientific) was used to observe cell changes. Using a conventional digital camera and adaptor, the cell status for each group was recorded at × 200 magnifications.

### Cell apoptosis analysis

Flow cytometry analysis was performed to detect apoptosis using fluorescein isothiocyanate (FITC) Annexin V Apoptosis Detection Kit Ι, following the manufacturer's instructions (BD Biosciences Pharmingen^TM^, Franklin Lakes, NJ, USA). Cells were seeded in 6-well plates (3 × 10^5^ cells /well) for 24 h. The cells were then trypsinized, and the single-cell suspension was added with FITC-Annexin V and propidium idodide (PI) for 15 min in the dark. Data was analyzed with FlowJo software (BD Biosciences). Stained cells were detected by a flow cytometer (Canto II, BD Biosciences). Early apoptosis is FITC Annexin V-positive and PI-negative, whereas late apoptosis is FITC Annexin V and PI double-positive 29.

### Migration and Invasion assay

Cell migration and invasion assays were performed using transwell chambers (8 μm pore size; BD Falcon, Bedford, MA, USA), which were coated without (migration assay) or with (invasion assay) Matrigel™ (Corning, Corning, NY, USA). Knocked-down *SVCT2* E264K-transfected HCT-15 cells (1 × 10^5^ cells) were suspended in 200 μl serum-free RPMI 1640 medium, and then added to the upper chambers. The lower chambers were filled with FBS-supplemented RPMI 1640 medium. After incubation for 18 h at 37°C in a 5% CO_2_ humidified incubator, cells were fixed with methanol, stained with hematoxylin and eosin (H&E), and then rinsed in distilled water, until the water was colorless. The cells in the inner chamber were removed with a cotton swab and cells attached to the bottom side of the membrane were counted in five random fields, per well, at × 200x magnification. Each experiment was performed in triplicate.

### Statistical analysis

Statistical analyses were performed using SigmaStat software v12 (Systat Software Inc., San Jose, CA, USA). Data were presented as means ± standard deviations (SDs) from 3 independent experiments. Significance of statistical analysis was analyzed using the Student's t-test. *p*<0.05 was considered statistically significant.

## Results

### SVCT2 E264K is overexpressed in CRC

**Figure 1:**
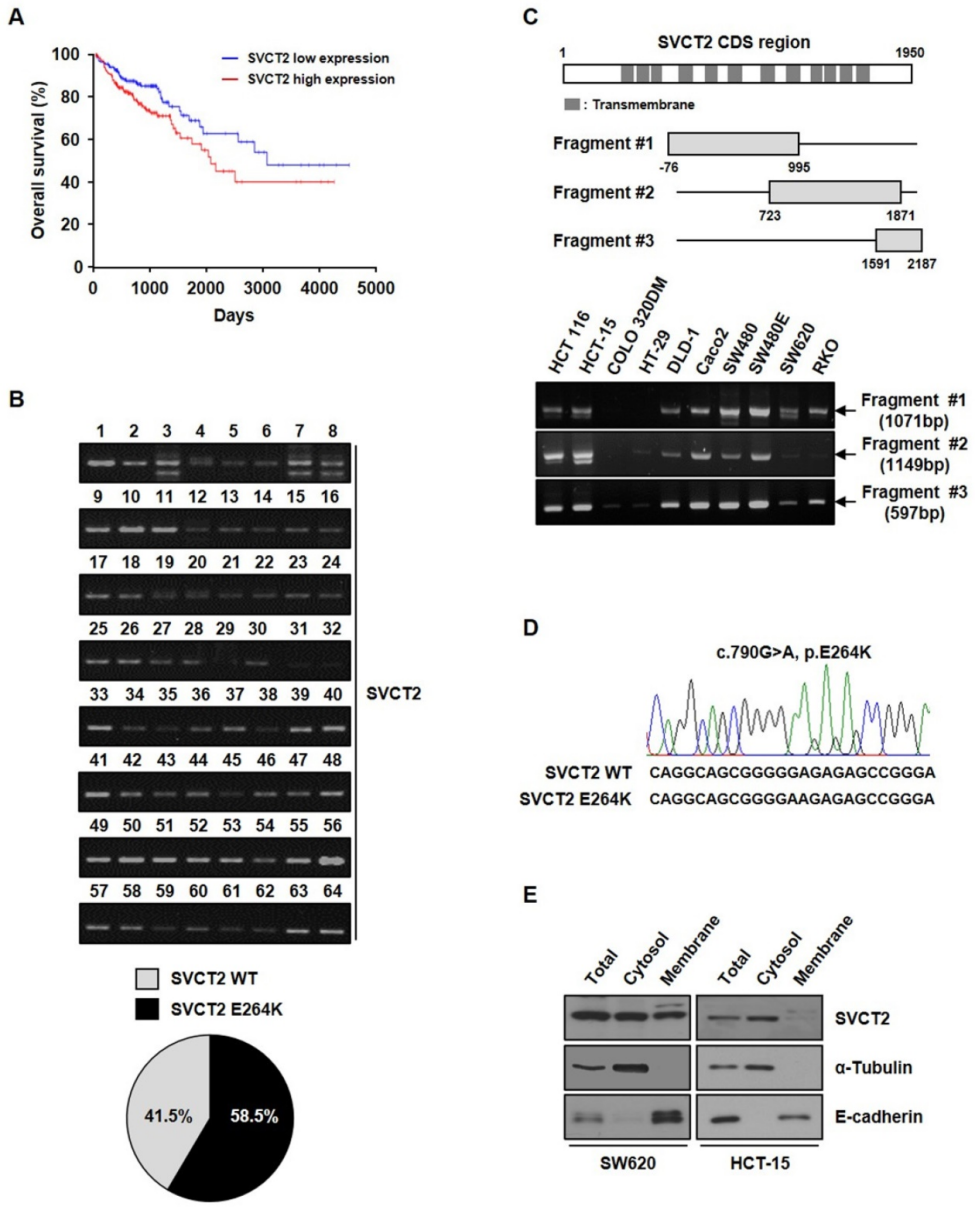
** Overexpression of *SVCT2* E264K in CRC.** (A) Overall survival analysis showed that *SVCT2* low expression (n=81) has a better prognosis than *SVCT2* high expression(n=81). *p*=0.038, HR(high)=1.8, p(HR)=0.041. (B) Expression of *SVCT2* E264K in 64 CRC patients using RT-PCR methods. (C) *SVCT2* levels were confirmed in 10 human CRC cell lines. (D) Expression of *SVCT2* E264K was analyzed by sequencing in SW620 and HCT-15. (E) SVCT2 localized to the membrane and cytosol in wild-type SVCT2-expressing SW620 cells , while HCT15 cells expressed *SVCT2* E264K only in the cytosol.

Kaplan-Meier Plotter survival curves showed that the overall survival was significantly longer in patients with lowly expressed *SVCT2*, compared to patients with highly expressed *SVCT2* (Fig. 1A). To identify target molecules for CRC patients, we performed RT-PCR using *SVCT2* E264K sequences and specific primers. We confirmed *SVCT2* E264K expression by DNA sequence analysis, in 58.5% of CRC patient tissues (Fig. 1B), suggesting that mutant SVCT2 E264K is a potential novel target for CRC. Therefore, to investigate the biological roles of *SVCT2* in the initiation and progression of CRC, we first examined its expression in human CRC cell lines HCT 116, HCT-15, COLO 320DM, HT-29, DLD-1, Caco2, SW480, SW480E, SW620 and RKO (Fig. 1C). As a result of sequencing analysis of the cell lines, it was found that SW620 was the *SVCT2* wild-type and HCT-15 was the *SVCT2* E264K type (Fig. 1D). In the immunohistochemistry data from the Human Protein Atlas, CRC patient's samples showed that SVCT2 expression is located in the cell membrane and cytoplasm (Supple. 1). Isolation of cytoplasmic and membrane fractions, from SW620 cells, showed SVCT2 to be present in both the cytosolic and membrane components, whereas *SVCT2* E264K, in HCT-15 cells, was restricted to the cytoplasm, rather than the membrane fraction (Fig. 1E). These results suggest that wild-type SVCT2 and SVCT2 E264K could serve different functions.

### SVCT2 E264K promotes cell proliferation by upregulating p-Akt

**Figure 2:**
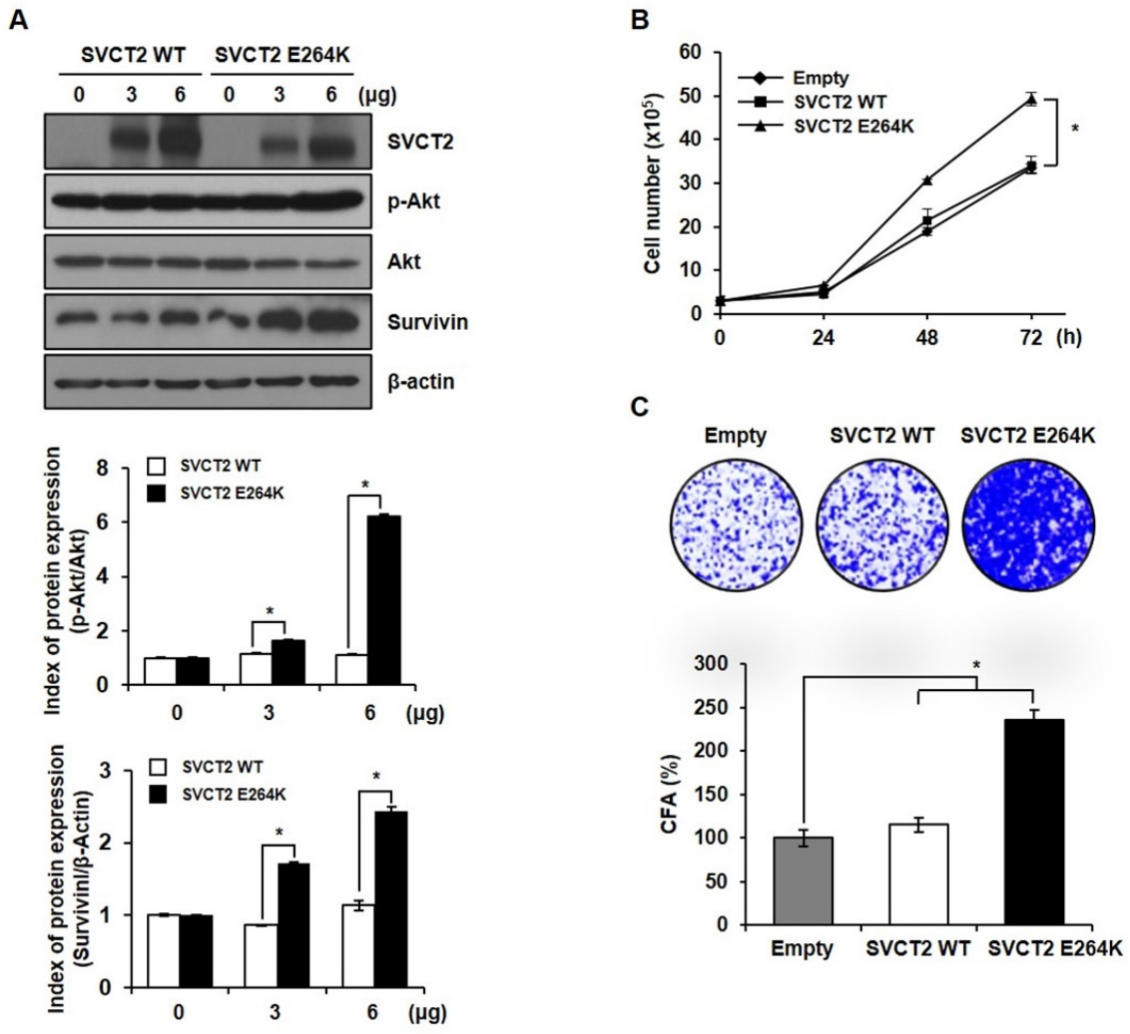
** The effects of *SVCT2* E264K on cell proliferation.** (A) Expression of p-Akt and Survivin was evaluated by western blotting in HEK293T cells. Cellular proliferation of *SVCT2* WT and *SVCT2* E264K-overexpressing cells analyzed using (B) trypan blue assay staining and (C) colony formation assay. *p<0.05 indicates significantly different from control group.

To determine whether SVCT2 E264K expression can accelerate the growth rate of cells, we confirmed the expression of a cell proliferation related marker. As a result, it was confirmed the expression of proto-oncoprotein p-Akt and Survivin was increased by *SVCT2* E264K (Fig. 2A). Also, *SVCT2* E264K showed greatly increased cell proliferation by trypan blue staining and colony forming assays, compared to wild-type SVCT2 (Fig. 2B and 2C). These results suggest that *SVCT2* E264K associates with pathogenic capacity in CRC.

### Knockdown of SVCT2 E264K suppresses CRC proliferation

**Figure 3:**
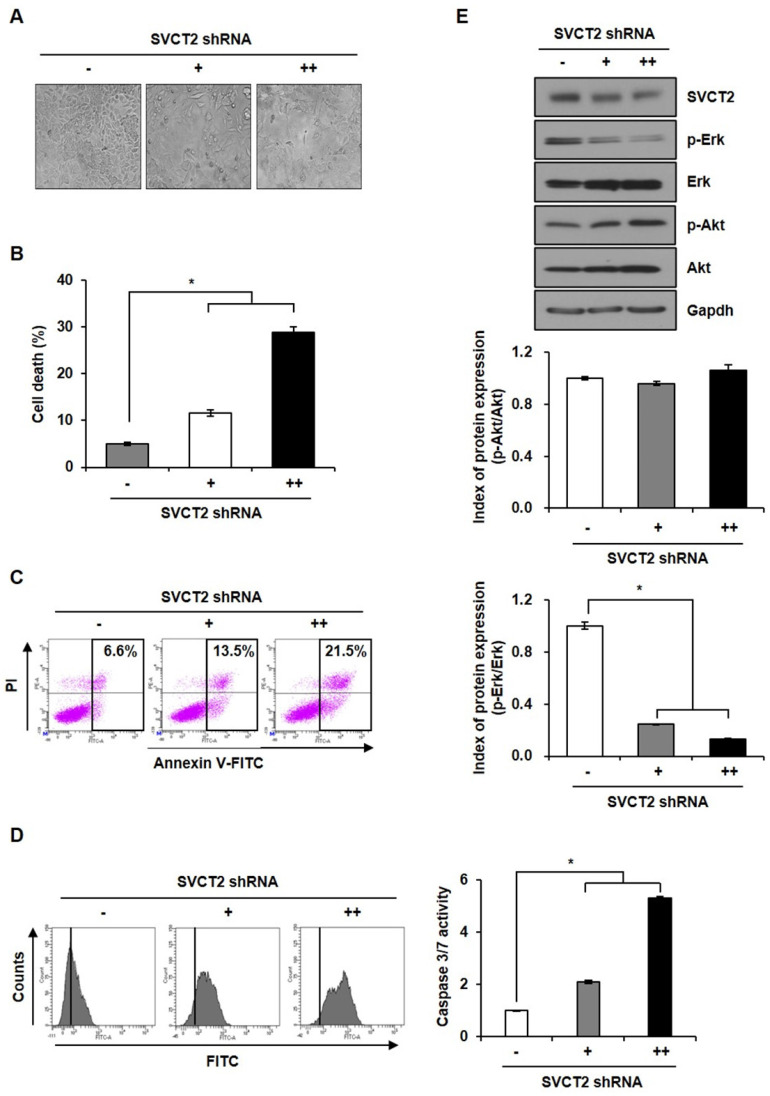
** The effects of *SVCT2* E264K knockdown on cell apoptosis.** (A) Following *SVCT2* E264K knockdown, as achieved by shRNA antagonism, HCT-15 cells were photographed under a light microscope. Scale bar, 100 μm. (B) Cell death after *SVCT2* E264K knockdown was confirmed by (B) trypan blue staining and (C) FITC-annexin V and PI staining (D) caspase 3/7 activity. (E) Expression of p-Erk and p-Akt was shown by western blotting in HCT-15 cells. *p<0.05; significantly different from control group.

To study the role of SVCT2 E264K, we investigated morphology changes following *SVCT2* E264K knockdown. Under microscopic observation, control HCT-15 cells displayed a round or polygonal shapes, and close cell-cell proximity, reminiscent of cellular tight-junctions. *SVCT2* E264K-knocked down HCT-15 cells, by contrast, dissociated from one another (Fig. 3A). Next, trypan blue staining revealed that SVCT2 E264K deficiency significantly increased cell death, implying that upregulation of the *SVCT2* E264K facilitates growth or survival of cancer cells (Fig. 3B). In addition, we analyzed the type of cell death in *SVCT2*-depleted cells by flow cytometry. FITC-conjugated Annexin V and PI double staining assay both showed that *SVCT2* E264K knockdown increases both early (Annexin V^+^/PI^-^) and late (Annexin V^+^/PI^+^) apoptosis (Fig. 3C). And caspase3/7 activity was significantly increased in *SVCT2* E264K knockdowned-cells (Fig. 3D). Erk 1/2 and Akt pathway are also important for cell proliferation 30, 31, and consistently, we observed that Akt activation (i.e., self-phosphorylation) was not influenced by *SVCT2* E264K knockdown. However, Erk 1/2 phosphorylation was definitively downregulated in these cells (Fig. 3E). These data imply that *SVCT2* E264K is strongly involved in CRC cell progression via inhibition of cell growth pathways.

### Knockdown of SVCT2 E264K inhibits cell migration and invasion

**Figure 4:**
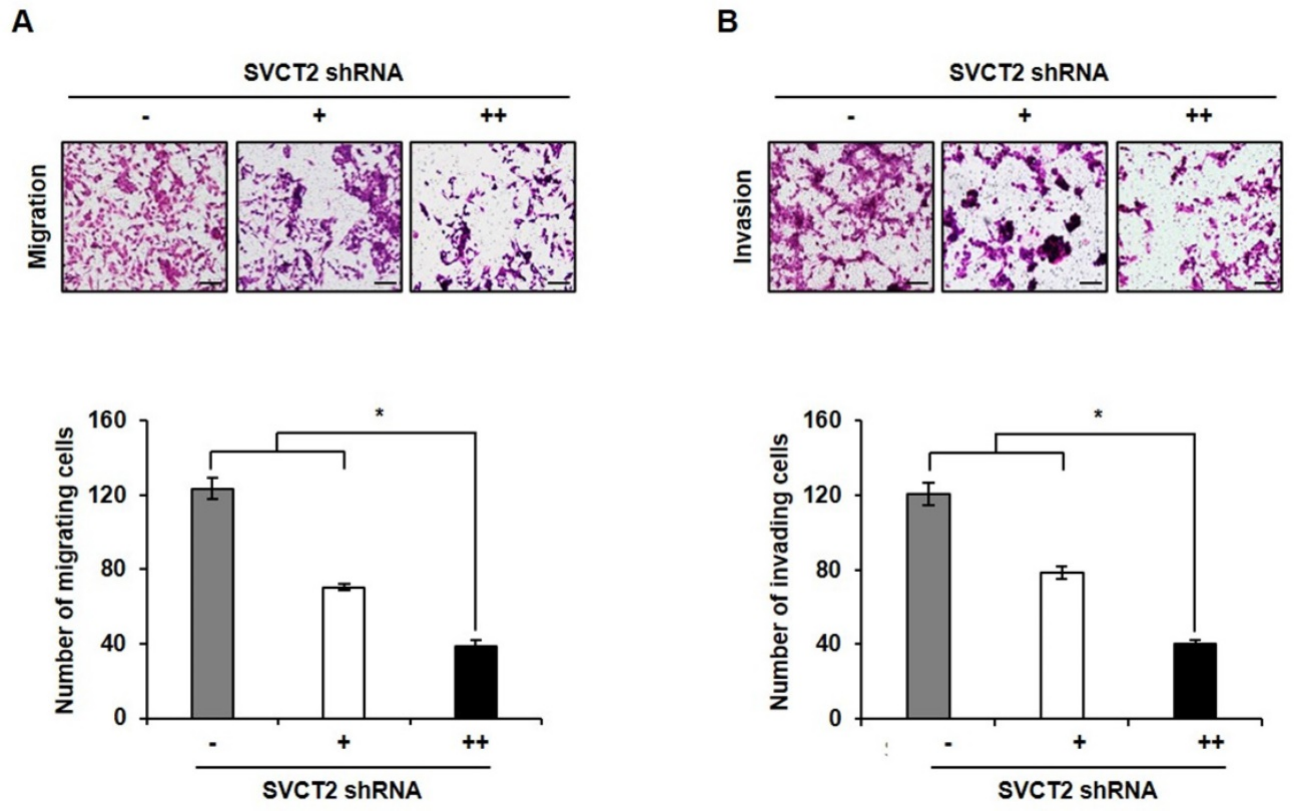
** The effects of *SVCT2* E264K knockdown on cell migration and invasion.** (A) Migration of SVCT2 E264K knockdown HCT-15 cells was assessed using uncoated transwell chambers. (B) Invasion of SVCT2 E264K knockdown HCT-15 cells was confirmed with Matrigel™-coated transwell chambers. Scale bar: 100 μm. *p<0.05 indicate significantly different from control group.

To evaluate whether knockdown of *SVCT2* modulated cell motility, we performed transwell invasion assays on *SVCT2* E264K-expressing HCT-15 cells. After knockdown of *SVCT2* E264K, cell migration ability was significantly inhibited (Fig. 4A). Consistently, invasion assays likewise indicated that knockdown of *SVCT2* E264K decreased cell invasiveness (Fig. 4B). These results strongly suggest that knockdown of *SVCT2* E264K inhibits CRC tumor progression.

## Discussion

SVCT2 is a high-affinity vitamin C transporter that regulates plasma-to-tissue accumulation of ascorbate, with *SVCT2* expressed ubiquitously in cells. Moreover, the SVCT2 transporter is highly expressed in the brain, where it is necessary for maintaining the high ascorbate levels required for proper brain function and survival 32, 33. In the human bronchial epithelium, SVCT2 protein expression inversely correlates with ascorbate concentration, in the respiratory tract-lining fluid 34. Also, mutant or wild-type SVCT2 protein was readily detected in Lewis lung tumors grown in ascorbate-dependent mice, and as predicted, SVCT2 protein levels varied over time, following a single high-dose ascorbate injection, although its association with tumor ascorbate levels is complex 35. In cancer, as the key protein responsible for vitamin C uptake in the liver, SVCT-2 plays crucial roles in regulating sensitivity to ascorbate-induced cytotoxicity 36.

In this study, however, we found that high levels of SVCT2 associated with poor survival in CRC, indicating that mutant or wild-type *SVCT2* expression could respectively, positively or negatively affect tumor growth. In in silico analysis, *SVCT2* is frequently mutated, and has a high pathogenic score (0.98), according to the Functional Analysis through Hidden Markov Models (FATHMM) (PMID: 23033316). It has previously been reported that CRC is a genetically heterogeneous disease by the sequential accumulation of multiple mutations. Specifically, our results showed expression of mutant *SVCT2* E264K in many CRC patients and cells. This genetic mutation can alter the subcellular localization of the SVCT2 protein 37, as we observed that wild-type SVCT2 largely localized to the cytoplasm and membrane, while mutant *SVCT2* E264K accumulated solely in the cytoplasm.

To further confirm the role of *SVCT2* E264K, we assessed whether wild-type *SVCT*, or its mutant, might modulate cell growth. These results confirmed that SVCT2 E264K upregulates the expression of p-Akt and Survivin proteins, both associated with cell proliferation, compared to SVCT2 wild-type, and that SVCT2 E264K promotes cell proliferation and colony-forming ability. These results indicate that SVCT2 E264K associates with acquired oncogenic potential. It has also been demonstrated that genetic mutations or DNA methylation lead to inactivation of functional tumor suppressor genes 38. Moreover, *SVCT2* E264K knockdown leads to increased cellular apoptosis and altered cell morphology. The SVCT2 E264K-expressing HCT15 cells displayed a round or polygonal shape, and close cell-cell proximity, reminiscent of cellular tight-junctions. By contrast, *SVCT2* E264K knockdown cells became dissociated from one another. This change in morphology is suggestive of the epithelial-to-mesenchymal transition (EMT). EMT refers to a global cellular and molecular transition in which polarized epithelial cells gain mesenchymal properties, allowing them to migrate, which plays a vital role in local invasion, and metastatic dissemination, during malignancy 39. Our results suggest that *SVCT2* E264K knockdown suppresses cell migration and invasion.

In summary, *SVCT2* E264K can influence tumor progression in CRC, and also is a promising potential biomarker for cancer diagnosis and prognosis. We will investigate further studies on which factors cause SVCT2 mutation. The present study provides novel insight into *SVCT2*-related cancers, and it is reasonable for us to conclude that *SVCT2* E264K knockdown inhibits CRC cell proliferation, invasion and migration. Therefore, this mutant protein could serve as a potential therapeutic target and prognostic predictor, for CRC patients.

## Supplementary Material

Supplementary figures.
