# Supplementary material for: Mutation SVCT2 promotes cell proliferation, invasion and migration in colorectal cancer
Source: J Cancer. 2021 Jul 6;12(18):5385–93. doi: 10.7150/jca.57463 (PMC8364649; doi:10.7150/jca.57463)
Supplement: Supplementary file 1 — Supplementary figures. [file jcav12p5385s1.pdf]

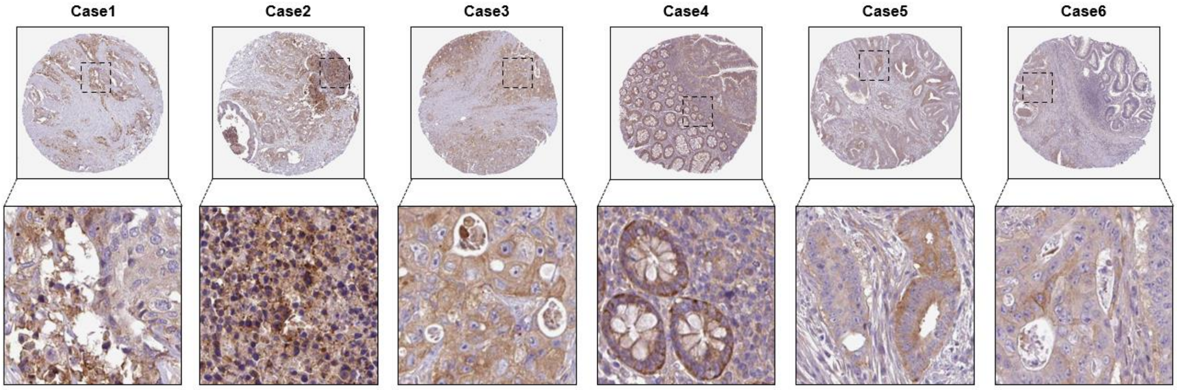

Supplementary figure 1. The representative protein expression of SVCT2 in colon cancer (case1-4) and rectal cancer (case 5-6) from the immunohistochemistry data were analyzed from the Human Protein Atlas (http://www.proteinatlas.org/).

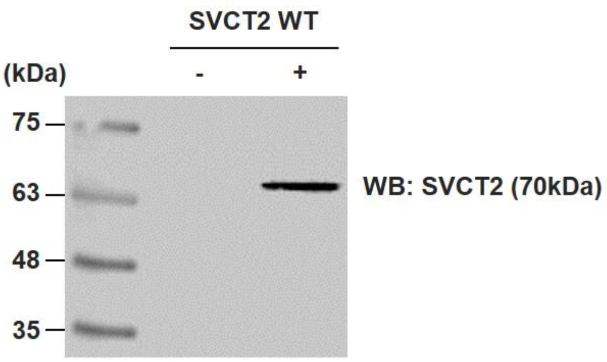

Supplementary figure 2. The protein expression of SVCT2 in HEK293T cells.
